# The Relationship Between Plasma DPP4 Activity to BDNF Ratio and Mild Cognitive Impairment in Elderly Population With Normal Glucose Tolerance

**DOI:** 10.3389/fnagi.2019.00033

**Published:** 2019-03-04

**Authors:** Liuping Xiao, Bo Ge, Xu Chen, Bo Chen, Linyuan Qin, Xueping Hu, Haidong Pan, Yujie Chen, Li Tian, Yun Gao, Tianpeng Zheng

**Affiliations:** ^1^Department of Endocrinology and Metabolism, The Second Affiliated Hospital of Guilin Medical University, Guilin, China; ^2^Department of Urology, The Second Affiliated Hospital of Guilin Medical University, Guilin, China; ^3^College of Pharmacy, Guilin Medical University, Guilin, China; ^4^Department of Human Anatomy, Southwest Medical University, Luzhou, China; ^5^Department of Epidemiology and Health Statistics, Guilin Medical University, Guilin, China; ^6^Department of Endocrinology and Metabolism, West China Hospital, Chengdu, China; ^7^Center of Diabetic Systems Medicine, Guangxi Key Laboratory of Excellence, Guilin Medical University, Guilin, China

**Keywords:** mild cognitive impairment, DPP4 activity to BDNF ratio, oxidative stress, inflammation, normal glucose tolerance

## Abstract

**Objective:** Since decreased brain-derived neurotrophic factor (BDNF) and increased dipeptidyl peptidase-4 (DPP4) activity have both been implicated in the pathogenesis of mild cognitive impairment (MCI), the aim of our study was to evaluate the association of MCI with plasma DPP4 activity to BDNF ratio (DBR) in an elderly population with normal glucose tolerance.

**Methods:** We cross-sectionally measured C-reactive protein, interleukin-6, nitrotyrosine, 8-iso-PGF2a, DPP4 activity BDNF and calculated the DBR in a total of 1,066 elderly participants in China. MCI was determined by the Montreal Cognitive Assessment and finally confirmed by neurologists.

**Results:** An inverse correlation was found between DPP4 activity and BDNF (*r* = -0.456, *P* < 0.001) and this inverse correlation was partly mediated by nitrotyrosine and 8-iso-PGF2a. Across rising quartiles of DBR, nitrotyrosine, 8-iso-PGF2a, C-reactive protein and interleukin-6 progressively increased, whereas the Montreal Cognitive Assessment score progressively decreased. Subjects in the lowest quartile of BDNF and highest quartiles of DBR and DPP4 activity, had higher MCI risk compared with subjects in the highest quartile of the BDNF and lowest quartiles of DBR and DPP4 activity, respectively (all *P* < 0.05). The odds ratio for MCI became more pronounced with decreased BDNF and increased DPP4.

**Conclusion:** In conclusion, a negative correlation was found between DPP4 activity and BDNF, and this negative correlation was partly mediated by oxidative stress, not inflammation. The DBR was positively associated with MCI and thus may be used as a novel risk biomarker for MCI in an elderly population with normal glucose tolerance.

## Introduction

Mild cognitive impairment (MCI) is a transitional step between normal cognition and dementia, in which patients have objective disturbances and cognitive complaints on cognitive examinations, but in which their ability to carry out daily life activities are not affected (Petersen et al., 2009; Ooi et al., 2011). Once MCI becomes more advanced, pharmacological interventions targeted at dementia are unlikely to delay further cognitive impairment (Zheng et al., 2016b; Chen et al., 2017), consequently, exploring the risk factors of MCI and its development mechanism is of great significance for the reduction of cognitive morbidity.

Dipeptidyl peptidase-4 (DPP4) is a serine exopeptidase belonging to the S9b DPP family that exists as a membrane-anchored cell surface protein or circulates as a soluble form in the peripheral circulation (Zheng et al., 2017, 2018b). Our previous data and other evidence in human or animals studies all proved that higher levels of DPP4 resulted in the enhancement of inflammation and oxidative stress, both of which have been implicated in the pathophysiology of cognitive decline (Ishibashi et al., 2013; Zheng et al., 2015a,b,c, 2018b). More importantly, our previous data demonstrated that plasma DPP4 activity was significantly and independently associated with MCI in subjects with normal glucose tolerance and the mechanisms may be explained by the mutual influence between oxidative stress and DPP4 (Chen et al., 2017). The BDNF, a member belonging to the neurotrophic superfamily of growth factors, plays a pivotal role in neuronal survival and repair, dendrite pruning and synaptic plasticity maintenance (Huang and Reichardt, 2001). A meta-analysis study indicated that MCI patients were accompanied by decreased circulating levels of BDNF, suggesting a link between reduced BDNF and the increased risk of MCI (Qin et al., 2017). Since increased circulating levels of DPP4 activity and decreased BDNF might both have been implicated in the pathophysiology of MCI, it is reasonable to speculate that BDNF might be inversely related to DPP4 activity and DBR might be considered as a novel biomarker for MCI in the elderly population with normal glucose tolerance. However, no study has ever investigated the relationship between BDNF and DPP4 activity or the feasibility of identifying DPP4 activity in the BDNF ratio as a novel biomarker for MCI in subjects with normal glucose tolerance.

Therefore, the aim of this study was to determine (1) whether plasma DPP4 activity is inversely related to, (2) what factors might mediate this inverse relationship, (3) whether DBR could be considered as a novel biomarker for MCI. Previous studies have found significant correlations between hyperglycemia and DPP4 activity, BDNF and cognitive impairment (Krabbe et al., 2007; Luchsinger et al., 2007; Zheng et al., 2014), to exclude the possible influences of hyperglycemia on DPP4 activity, BDNF and cognitive function. Our study was conducted in a non-diabetic population.

## Materials and Methods

### Subjects

The study participants included 1066 elderly non-diabetic subjects aged 60 years or older, who visited the Medical Examination Department of the Affiliated Hospital of Guilin Medical University, between 2013 and 2016. Subjects were excluded if they met any of the following criteria: (1) presence of diseases comprising of hyperglycemia, head trauma, hypertensive crisis, malignancy, hypothyroidism, non-alcoholic fatty liver disease, chronic or acute inflammatory diseases, dementia, heart, liver, respiratory, and kidney dysfunction, (2) history of psychological disturbances, visual/auditory disorders and central nervous system disease that might result in dementia, (3) those who had taken medication affecting circulating levels of DPP4 activity or cognitive function within one year before enrollment, (4) drug or alcohol abuse. Our study was approved by the Drugs and Medical Apparatus Ethics Committee at the Affiliated Hospital of Guilin Medical University, and all participants provided signed written informed consent (Trial Registration Number: ChiCTR-EPC-14005273).

### Data Collection

Demographic and clinical data were recorded in a standardized interview by trained staff. Venous blood specimen was collected from fasting participants to measure circulating levels of CRP, IL-6, 8-iso-PGF2a, nitrotyrosine, BDNF, and DPP4 activity. The plasma levels of BDNF were determined using an ELISA kit (R&D, United States), BDNF concentrations were obtained against a standard curve and expressed as ng/ml. DPP4 activity, 8-iso-PGF2a, nitrotyrosine, CRP, and IL-6 were assayed as previously described (Zheng et al., 2014, 2015a). MCI was determined by the MoCA and finally confirmed by neurologists (Albert et al., 2011). MoCA tests eight cognitive domains, visual–spatial ability, attention, executive function, immediate memory, delayed memory, language, abstraction, calculation, and orientation-for a maximum total score of 30. The normal MoCA score is ≥26, with 1 point added if the subject has fewer than 12 years of formal education (Nasreddine et al., 2005; Zheng et al., 2016b).

### Statistical Analysis

Statistical analyses were performed with SPSS version 16.0. Data were presented as proportions, frequencies, median (interquartile range) or mean ± standard deviation. We used the *t*-test, chi-square and ANCOVA to compare clinical and biochemical parameters. ANCOVA was conducted to compare continuous variables across DBR quartiles after adjusting for age, gender, and BMI. The relationships between parameters were investigated with partial correlation analysis and multivariate logistic regression analysis.

Mediation analysis was conducted as follows: (1) *Y* = cX + e1 (2) *M* = aX + e2 (3) *Y* = c’X + bM + e3, X represents DPP4 activity, *Y* represents BDNF, *M* represents the mediator, a represents the regression coefficient for the relationship between DPP4 activity and mediator, b represents the regression coefficient for the relationship between mediator and BDNF, c represents the regression coefficient for the relationship between DPP4 and BDNF, and c’ represents the direct effect of DPP4 on BDNF after controlling for the indirect effect. A Sobel Test was conducted to further test mediation. An indirect ratio was calculated to estimate the strength of mediation: ([a^∗^b]/c) (Yang et al., 2018; Zheng et al., 2018a).

## Results

### Clinical and Laboratory Characteristics

Table 1 shows the characteristics of all participants according to DBR quartiles. Participants with higher DBRs were relatively old (*P* < 0.05), with a lower BDNF and MoCA score and higher 8-iso-PGF2a, nitrotyrosine, CRP, IL-6 and DPP4 activity (all *P* < 0.05). Compared with controls, subjects with MCI had a lower BDNF and MoCA score and higher 8-iso-PGF2a, nitrotyrosine, DPP4 activity and a DBR (Supplementary Table 1). Compared with male participants, female participants had higher BDNF levels (*P* < 0.01) (2.36 ± 1.11 ng/ml in men vs. 2.65 ± 1.07 ng/ml in women) (Supplementary Figure S2).

**Table 1:** Characteristics of study participants according to quartiles of the DPP4 activity to BDNF ratio (DBR).

Characteristics	Total	Q1	Q2	Q3	Q4	*P*-value
	(*n* = 1066)	(*n* = 267)	(*n* = 266)	(*n* = 267)	(*n* = 266)	
		<4.01	4.01-6.86	6.87-12.28	>12.28	
DBR	10.06 ± 9.65	2.68 ± 0.83	5.34 ± 0.82	9.25 ± 1.58	23.02 ± 11.12	<0.001
Age(years)	68.9 ± 5.4	68.7 ± 5.1	68.2 ± 5.6	69.1 ± 5.4	69.5 ± 5.5	0.044
Percent men (%)	41.8	39.7	38.0	41.2	48.5	0.071
Body mass index (kg/m^2^)	23.3 ± 3.7	22.6 ± 3.6	23.3 ± 3.6	23.4 ± 3.7	23.9 ± 3.9	0.001
Current smoking (%)	20.2	18.0	17.7	20.2	24.8	0.145
Habitual alcohol drinking (%)	17.2	14.6	17.3	16.1	20.7	0.290
Leisure-time physical activity (%)	56.8	58.4	57.5	54.7	56.8	0.842
Education level						0.398
≤Primary school	49.0	44.6	50.8	49.8	50.8	
Middle school	42.6	47.9	40.2	43.1	39.1	
≥High school	8.4	7.5	9.0	7.1	10.1	
Annual income, RMB						0.227
≤5000	4.8	3.7	4.5	5.2	5.6	
5000–30000	45.4	45.0	44.0	51.7	41.0	
>30000	49.8	51.3	51.5	43.1	53.4	
Statin use (%)	11.3	10.5	9.8	12.0	12.8	0.681
NSAID use (%)	6.2	5.2	5.3	6.7	7.5	0.625
Cardiovascular disease (%)	7.9	7.5	6.8	6.7	10.5	0.314
SBP^a^	120 ± 19	119 ± 16	119 ± 18	122 ± 20	121 ± 22	0.488
DBP^a^	71 ± 9	71 ± 8	71 ± 8	70 ± 9	70 ± 10	0.503
TG (mmol/L)^a^	1.45 (1.10,1.98)	1.31 (0.99,1.84)	1.35 (0.99,1.91)	1.50 (1.17,2.01)	1.61 (1.27,2.15)	0.002
TC (mmol/L)^a^	5.14 ± 0.95	5.04 ± 0.88	5.16 ± 0.96	5.18 ± 0.99	5.16 ± 0.98	0.499
LDL-C (mmol/L)^a^	3.05 ± 0.89	2.99 ± 0.90	3.11 ± 0.90	3.08 ± 0.87	3.00 ± 0.90	0.508
HDL-C (mmol/L)^a^	1.40 ± 0.37	1.39 ± 0.40	1.44 ± 0.38	1.41 ± 0.36	1.36 ± 0.37	0.268
IL-6 (pg/ml)^a^	1.36 (1.11,1.60)	1.31 (1.07,1.48)	1.29 (1.03,1.46)	1.38 (1.16,1.64)	1.53 (1.23,1.90)	<0.001
CRP (mg/L)^a^	1.16 (0.94,1.34)	1.09 (0.86,1.25)	1.15 (0.92,1.29)	1.19 (0.97,1.41)	1.23 (0.97,1.58)	<0.001
Nitrotyrosine (μmol/L)^a^	0.39 ± 0.12	0.34 ± 0.10	0.36 ± 0.10	0.40 ± 0.12	0.47 ± 0.13	<0.001
8-iso-PGF2a (pg/mL)^a^	42.5 ± 10.8	36.3 ± 9.6	41.1 ± 9.7	43.6 ± 10.7	49.2 ± 8.8	<0.001
DPP4 activity (nmol/min/ml)^a^	18.1 ± 8.1	9.7 ± 3.5	15.0 ± 3.9	20.1 ± 4.7	27.5 ± 6.3	<0.001
BDNF (ng/ml)^a^	2.53 ± 1.10	3.70 ± 0.90	2.84 ± 0.76	2.21 ± 0.53	1.35 ± 0.47	<0.001
MoCA score^a^	26.9 ± 2.4	27.9 ± 2.0	27.2 ± 2.2	26.8 ± 2.1	25.8 ± 2.7	<0.001

*Data were expressed as means ± standard deviation, median (interquartile range), or percentage for normally distributed continuous various, abnormally distributed continuous variables (TG, IL-6, CRP), and categorical variables, respectively. Cigarette smoking was defined as having smoked at least 100 cigarettes in one’s lifetime. Regular leisure-time physical activity was defined as participation in ≥30 min of moderate or vigorous activity per day at least 3 days per week. ^*a*^Adjusted for age, gender, and BMI*.

### The Relationships Between DBR and Other Parameters

Partial correlation analyses revealed that (1) DPP4 was inversely related to BDNF in all participants, (2) 8-iso-PGF2a, nitrotyrosine, CRP, and IL-6 were all positively correlated with DBR and DPP4 activity and negatively with BDNF, (3) BDNFs were positively associated, and DPP4 and DBRs negatively, with the MoCA score (Table 2).

**Table 2:** Correlations between DPP4 activities, BDNF, and DBR vs. metabolic parameters and MoCA score.

	DPP4 activity^a^		BDNF^a^		DBR^a^	
	r	p	r	p	r	p
DPP4 activity	-	-	-0.456	<0.001	0.750	<0.001
BDNF	-0.456	<0.001	-	-	-0.688	<0.001
IL-6	0.299	<0.001	-0.137	<0.001	0.215	<0.001
CRP	0.225	<0.001	-0.129	<0.001	0.218	<0.001
nitrotyrosine	0.304	<0.001	-0.415	<0.001	0.362	<0.001
8-iso-PGF2a	0.328	<0.001	-0.471	<0.001	0.407	<0.001
MoCA score	-0.306	<0.001	0.209	<0.001	-0.254	<0.001

^a^*P-value determined by partial correlation analysis with respect to the DPP4 activity, BDNF, and DBR adjusted for age, BMI, gender, education level, statin use and NSAID use*.

### Mediation Analysis

**Figure 1:**
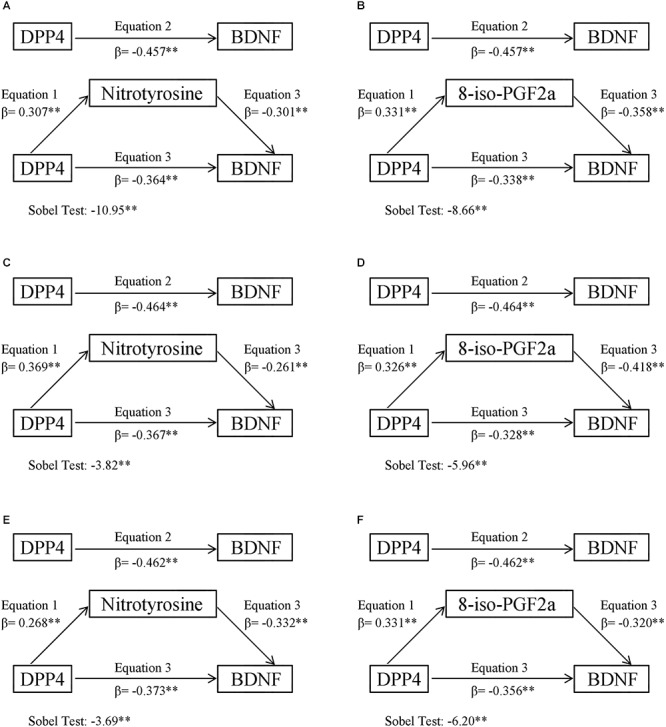
Oxidative stress mediation models of the relationship between plasma DPP4 activity and BDNF. **(A)** Nitrotyrosine mediation models of the relationship between plasma DPP4 activity and BDNF in all participants. **(B)** 8-iso-PGF2a mediation models of the relationship between plasma DPP4 activity and BDNF in all participants. **(C)** Nitrotyrosine mediation models of the relationship between plasma DPP4 activity and BDNF in men. **(D)** 8-iso-PGF2a mediation models of the relationship between plasma DPP4 activity and BDNF in men. **(E)** Nitrotyrosine mediation models of the relationship between plasma DPP4 activity and BDNF in women. **(F)** 8-iso-PGF2a mediation models of the relationship between plasma DPP4 activity and BDNF in women. ^∗^*P* < 0.05, ^∗∗^*P* < 0.01.

Figure 1A,B presented the mediating role of 8-iso-PGF2a and nitrotyrosine in the negative correlation between DPP4 and BDNF in all subjects, in the first model, 8-iso-PGF2a and nitrotyrosine were both positively correlated with DPP4 activity (*P* < 0.01), in the second model, BDNF was inversely correlated with DPP4 activity (*P* < 0.01), in the third model, DPP4 activity, nitrotyrosine or 8-iso-PGF2a were all inversely correlated with BDNF (*P* < 0.01). A Sobel test revealed that 8-iso-PGF2a and nitrotyrosine both had an indirect effect, representing 20.4 and 26.0% of the total effect on the correlation between BDNF and DPP4 activity, respectively. When the participants were further divided into two subgroups according to gender, the results of the mediating role of oxidative stress parameters in the inverse correlation between DPP4 activity and BDNF in male and female participants showed similar results (Figure 1C–F), such that 8-iso-PGF2a and nitrotyrosine were partial mediators in both male and female subjects, the percentages of the indirect effect mediated by 8-iso-PGF2a and nitrotyrosine were 22.9 and 19.3% in women and 29.3 and 20.8% in men, respectively. The mediating role of inflammatory markers in the negative correlation between BDNF and DPP4 activity were also tested (Supplementary Figure S1), in the third model, both CRP and IL-6 were not significantly correlated with BDNF, therefore, inflammation markers did not play a mediating role in the negative relationship between DPP4 activity and BDNF.

### Associations Between DBR and MCI

**Figure 2:**
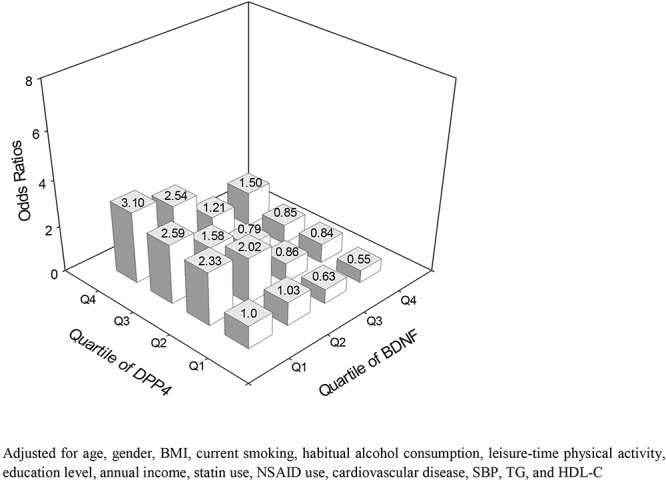
Adjusted ORs for MCI according to the quartiles of DPP4 activity and BDNF.

The prevalence of MCI in this study was 15.9%. The odds ratio for MCI were higher with decreasing BDNF quartiles and increasing DBR and DPP4 quartiles. The odds ratio for MCI in the highest quartiles of DBR, DPP4 and BDNF were as follows: 4.63 for DBR, 3.78 for DPP4 and 0.28 for BDNF (*P* < 0.01) (Table 3). The odds ratio for MCI became higher in subjects with lower BDNF and higher DPP4 activity (Figure 2).

**Table 3:** Logistic regression analysis of the association of DPP4 activity, BDNF, DBR, and MCI.

	Q1	Q2	Q3	Q4
DPP4 activity (nmol/ml/min)	<11.97	11.97-16.87	16.88-24.02	>24.02
MCI	21 (7.9%)	40 (15.0%)	39 (14.6%)	69 (25.9%)
Model 1	1	2.06 (1.18, 3.59) 0.011	2.00 (1.14, 3.50) 0.016	4.09 (2.42, 6.90) < 0.001
Model 2	1	1.88 (1.06, 3.33) 0.030	2.00 (1.12, 3.56) 0.018	3.78 (2.20, 6.49) < 0.001
BDNF (ng/ml)	<1.71	1.71-2.37	2.38-3.22	>3.22
MCI	70 (26.2%)	50 (18.7%)	26 (9.8%)	23 (8.6%)
Model 1	1	0.65 (0.43, 0.98) 0.039	0.31 (0.19, 0.50) < 0.001	0.27 (0.16, 0.44) < 0.001
Model 2	1	0.69 (0.45, 1.06) 0.091	0.31 (0.19, 0.51) < 0.001	0.28 (0.17, 0.48) < 0.001
DBR	<4.01	4.01-6.86	6.87-12.28	>12.28
MCI	21 (7.9%)	31 (11.7%)	39 (14.6%)	78 (29.3%)
Model 1	1	1.55 (0.86, 2.77) 0.143	2.00 (1.14, 3.51) 0.015	4.86 (2.90, 8.16) < 0.001
Model 2	1	1.52 (0.84, 2.74) 0.165	1.94 (1.10, 3.43) 0.023	4.63 (2.73, 7.88) < 0.001

*Model 1, crude model; Model 2, Model 1 + age + gender + BMI + current smoking + habitual alcohol consumption + leisure-time physical activity + education level + annual income + statin use + NSAID use + cardiovascular disease + SBP + TG + HDL-C*.

## Discussion

The novel findings of this study were that (1) circulating levels of BDNF were inversely correlated with DPP4 activity, (2) the inverse correlation between BDNF and DPP4 was partially mediated by oxidative stress, not inflammation (3) higher levels of DBR and DPP4 activity and lower levels of BDNF were positively related to the increased risk of MCI.

Previous researches suggested that decreased BDNF and increased DPP4 activities were found in the periphery circulation of patients with MCI (Zheng et al., 2016b; Qin et al., 2017), suggesting that BDNF and DPP4 activity might both play a pathogenetic role in the development of cognitive impairment. Consistently, our current study also found that MCI patients with normal glucose tolerance had lower BDNF and higher DPP4 activity in periphery circulation, compared with the controls. More importantly, an inverse correlation was found between BDNF and DPP4 activity in this study, since oxidative stress and inflammation have both been suggested to be linked to DPP4 and BDNF, we used mediation analyses to test whether this inverse correlation between BDNF and DPP4 activity was mediated by inflammation and oxidative stress. In this study, CRP and IL-6 were chosen as inflammatory markers and 8-iso-PGF2a and nitrotyrosine were chosen as oxidative stress parameters, respectively. The reason for this selection could be summarized as follows: (1) there is growing evidence linking cognitive function with proinflammatory markers, in particular the downstream CRP and the upstream cytokines IL-6 (Marioni et al., 2010; Tegeler et al., 2016), CRP and IL-6 were both found to be predictive of future cognitive impairment in previous studies (Weaver et al., 2002; Kuo et al., 2005). (2) 8-iso-PGF2a and nitrotyrosine have both been validated to accurately assess the level of oxidative stress in human studies (Ceriello et al., 2011, 2013).

Accumulated basic and clinical evidence have demonstrated that DPP4 dose-dependently increased oxidative stress production (Ishibashi et al., 2013; Zheng et al., 2015b). More importantly, previous studies have proved that DPP4 inhibitors, such as Alogliptin or Vildagliptin, suppressed oxidative stress in different animal models (Abdelsalam and Safar, 2015; Yisireyili et al., 2016; Aroor et al., 2017; Zhang et al., 2018). With regard to the association between oxidative stress and BDNF, oxidative stress has been proven to cause a decrease in BDNF expression in basic research, while the underlying mechanisms was partly attributed to the impairment of N-methyl-D-aspartate channel (Wu et al., 2004). In this study, our findings from mediation analyses were consistent with previous reports, that DPP4 activity were positively related, and BDNF negatively, to oxidative stress parameters in the linear regression model, furthermore, oxidative stress parameters 8-iso-PGF2a and nitrotyrosine both played a mediating role in the negative correlation between BDNF and DPP4 activity, with indirect effects contributing 20.4 and 26.0% of the total effect, respectively. Since a gender difference exists in BDNF levels in periphery circulation (Trajkovska et al., 2007), we further divided all participants into two subgroups according to gender, and mediation analysis showed that the percentages of oxidative stress-mediated indirect effects in men were higher than that in women, this result might be partly due to the fact that circulating levels of BDNF in men were lower than in women. In addition, a gender difference has also been reported for DPP4 activity (Durinx et al., 2001), Durinx et al. proved that plasma DPP4 activity in women was slightly lower than in men. However, in this study, we did not find a gender difference in plasma DPP4 activity (18.3 ± 8.4 nmol/min/ml in men vs. 17.9 ± 7.9 nmol/min/ml in women), this discrepancy might partly be attributed to differences in sample size, study design, medicine status, race and use of DPP4 activity kits. We cannot rule out the possibility that the higher percentages of oxidative stress-mediated indirect effects in men might be partly due to the higher plasma DPP4 activity in men as well.

The relationships between DPP4, BDNF and inflammation have also been extensively investigated. The proinflammatory effects of DPP4 and its underlying mechanism have been well addressed (Tian et al., 2010; Shah et al., 2011; Zhong et al., 2013; Zheng et al., 2015c; Zhong and Rajagopalan, 2015; Duan et al., 2017), consistently, throughout this study, and a positive relationship was found between IL-6, CRP and DPP4 activity. Results regarding the correlation between BDNF and inflammation were more complicated and inconsistent, on one hand, inflammation could lead to both an increase and a decrease in BDNF production at different organ tissues, on the other hand, BDNF might in turn exert pro- and anti-inflammatory influences (Papathanassoglou et al., 2015). The inconsistencies in previous studies regarding the relationship between inflammation and BDNF might be partly attributed to mutual interactions between BDNF and inflammation at different parts of the human body. In this study, partial correlation controlling for possible confounders proved a negative correlation between BDNF and inflammation markers, however, when both inflammation markers and DPP4 activity were simultaneously added in the linear regression model, no significant relationship was found between BDNF and inflammation markers, consequently, these results suggested that inflammation did not play a mediating role in the inverse correlation between BDNF and DPP4 activity.

Logistic regression analysis proved that participants in the lowest quartile of BDNF and the highest quartiles of DBR and DPP4 activity had the highest MCI risk, more importantly, the MCI risk in the lowest quartile of BDNF and the highest quartile of DPP4 activity were lower than that in the highest quartile of DBR, suggesting that the combination of BDNF and DPP4 activity, which presented as DBR, might be more suitable to serve as a risk biomarker for MCI than either BDNF or DPP4 activity alone. Based on these findings above, we extrapolated that increased DPP4 activity might result in the enhancement of oxidative stress, leading to a decrease in BDNF expression. This, in turn, impaired neuronal survival and synaptic plasticity maintenance and finally promoted the development of MCI. However, further studies are needed to clarify this speculation.

There were some limitations to our study, (1) since this study was a cross-sectional research, inferences about causalities are limited, (2) some unknown factors other than oxidative stress might also exist to mediate the negative correlation between BDNF and DPP4, (3) the presence of some unmeasured confounders may also have had some effects on the relationship between MCI and DBR limiting generalizations of this study, however, after adding a wide range of potential confounders in the statistical analyses, the strong relationship between DBR and MCI are unlikely to be an artifact, (4) we failed to discuss how other diseases may influence DBR. Previous studies have reported higher levels of DPP4 activity in patients with diabetes, obesity and depression (Zheng et al., 2014, 2016a; Nargis and Chakrabarti, 2018), in addition, diabetic and depressive patients both exhibited lower levels of BDNF in the circulation (Krabbe et al., 2007; Molendijk et al., 2014), therefore, whether DBR could be used as a novel risk biomarker for MCI in patients with diabetes, obesity and depression remains to be clarified in further research.

In conclusion, our current data supported the negative relationship between BDNF and DPP4 activity in MCI, from a clinical perspective, we extrapolated that this negative relationship was partly mediated by oxidative stress, not inflammation. In particular, DBR might be more suitable to serve as a risk biomarker for MCI than either BDNF or DPP4 activity alone, in an elderly non-diabetic population. However, because of the nature of this study, the feasibility of identifying DBR as a novel biomarker or even a therapeutic target for MCI still needs to be confirmed by future studies.

## Author Contributions

TZ, BG, LX, and XC conceptualized and designed the study. TZ, BG, BC, XC, LQ, XH, LX, HP, YC, and LQ performed the data acquisition and analysis, or interpreted the data. TZ, LX, LT, and YG drafted the manuscript. TZ, LT, and YG critically revised the manuscript for important intellectual content. LQ performed the statistical analysis. TZ obtained funding and supervised the study. All authors approved the final manuscript to be published and agreed to be accountable for all aspects of the work in ensuring that questions related to the accuracy or integrity of any part of the work are appropriately investigated and resolved.

## Conflict of Interest Statement

The authors declare that the research was conducted in the absence of any commercial or financial relationships that could be construed as a potential conflict of interest.

## Supplementary Material

FIGURE S1 Inflammation mediation models of the relationship between plasma DPP4 activity and BDNF **(A)** IL-6 mediation models of the relationship between plasma DPP4 activity and BDNF in all participants. **(B)** CRP mediation models of the relationship between plasma DPP4 activity and BDNF in all participants. **(C)** IL-6 mediation models of the relationship between plasma DPP4 activity and BDNF in men. **(D)** CRP mediation models of the relationship between plasma DPP4 activity and BDNF in men. **(E)** IL-6 mediation models of the relationship between plasma DPP4 activity and BDNF in women. **(F)** CRP mediation models of the relationship between plasma DPP4 activity and BDNF in women. ^∗^*P* < 0.05, ^∗∗^*P* < 0.01.

FIGURE S2 Comparison of BDNF levels between men and women. ^∗^*P* < 0.05, ^∗∗^*P* < 0.01.

TABLE S1 Characteristics of the 1066 participants by MCI.

## Abbreviations

ANCOVA: analysis of covariance
BDNF: brain-derived neurotrophic factor
BMI: body mass index
CRP: C-reactive protein
DBR: DPP4 activity to BDNF ratio
DPP4: dipeptidyl peptidase-4
IL-6: interleukin-6
MCI: mild cognitive impairment
MoCA: montreal cognitive assessment

## References

[B1] Abdelsalam R. M. Safar M. M. (2015). Neuroprotective effects of vildagliptin in rat rotenone Parkinson’s disease model: role of RAGE-NFkappaB and Nrf2-antioxidant signaling pathways. *J. Neurochem.* 133 700–707. 10.1111/jnc.13087 25752913

[B2] Albert M. S. Dekosky S. T. Dickson D. Dubois B. Feldman H. H. Fox N. C. (2011). The diagnosis of mild cognitive impairment due to Alzheimer’s disease: recommendations from the national institute on Aging-Alzheimer’s association workgroups on diagnostic guidelines for Alzheimer’s disease. *Alzheimers Dement.* 7 270–279. 10.1016/j.jalz.2011.03.008 21514249 PMC3312027

[B3] Aroor A. R. Habibi J. Kandikattu H. K. Garro-Kacher M. Barron B. Chen D. (2017). Dipeptidyl peptidase-4 (DPP-4) inhibition with linagliptin reduces western diet-induced myocardial TRAF3IP2 expression, inflammation and fibrosis in female mice. *Cardiovasc. Diabetol.* 16:61. 10.1186/s12933-017-0544-4 28476142 PMC5420102

[B4] Ceriello A. Esposito K. Testa R. Bonfigli A. R. Marra M. Giugliano D. (2011). The possible protective role of glucagon-like peptide 1 on endothelium during the meal and evidence for an “endothelial resistance” to glucagon-like peptide 1 in diabetes. *Diabetes Care* 34 697–702. 10.2337/dc10-1949 21273492 PMC3041210

[B5] Ceriello A. Novials A. Ortega E. Canivell S. La Sala L. Pujadas G. (2013). Vitamin C further improves the protective effect of glucagon-like peptide-1 on acute hypoglycemia-induced oxidative stress, inflammation, and endothelial dysfunction in type 1 diabetes. *Diabetes Care* 36 4104–4108. 10.2337/dc13-0750 24130351 PMC3836129

[B6] Chen B. Zheng T. Qin L. Hu X. Zhang X. Liu Y. (2017). Strong association between plasma dipeptidyl peptidase-4 activity and impaired cognitive function in elderly population with normal glucose tolerance. *Front. Aging Neurosci.* 9:247. 10.3389/fnagi.2017.00247 28798686 PMC5526854

[B7] Duan L. Rao X. Braunstein Z. Toomey A. C. Zhong J. (2017). Role of incretin axis in inflammatory Bowel disease. *Front. Immunol.* 8:1734. 10.3389/fimmu.2017.01734 29270177 PMC5723660

[B8] Durinx C. Neels H. Van der Auwera J. C. Naelaerts K. Scharpe S. De Meester I. (2001). Reference values for plasma dipeptidyl-peptidase IV activity and their association with other laboratory parameters. *Clin. Chem. Lab. Med.* 39 155–159. 10.1515/CCLM.2001.026 11341750

[B9] Huang E. J. Reichardt L. F. (2001). Neurotrophins: roles in neuronal development and function. *Annu. Rev. Neurosci.* 24 677–736. 10.1146/annurev.neuro.24.1.677 11520916 PMC2758233

[B10] Ishibashi Y. Matsui T. Maeda S. Higashimoto Y. Yamagishi S. (2013). Advanced glycation end products evoke endothelial cell damage by stimulating soluble dipeptidyl peptidase-4 production and its interaction with mannose 6-phosphate/insulin-like growth factor II receptor. *Cardiovasc. Diabetol.* 12:125. 10.1186/1475-2840-12-125 23984879 PMC3765742

[B11] Krabbe K. S. Nielsen A. R. Krogh-Madsen R. Plomgaard P. Rasmussen P. Erikstrup C. (2007). Brain-derived neurotrophic factor (BDNF) and type 2 diabetes. *Diabetologia* 50 431–438. 10.1007/s00125-006-0537-534 17151862

[B12] Kuo H. K. Yen C. J. Chang C. H. Kuo C. K. Chen J. H. Sorond F. (2005). Relation of C-reactive protein to stroke, cognitive disorders, and depression in the general population: systematic review and meta-analysis. *Lancet Neurol.* 4 371–380. 10.1016/S1474-4422(05)70099-95 15907742

[B13] Luchsinger J. A. Reitz C. Patel B. Tang M. X. Manly J. J. Mayeux R. (2007). Relation of diabetes to mild cognitive impairment. *Arch. Neurol.* 64 570–575. 10.1001/archneur.64.4.570 17420320

[B14] Marioni R. E. Strachan M. W. Reynolds R. M. Lowe G. D. Mitchell R. J. Fowkes F. G. (2010). Association between raised inflammatory markers and cognitive decline in elderly people with type 2 diabetes: the Edinburgh Type 2 diabetes study. *Diabetes* 59 710–713. 10.2337/db09-1163 19959761 PMC2828661

[B15] Molendijk M. L. Spinhoven P. Polak M. Bus B. A. A. Penninx B. W. J. H. Elzinga B. M. (2014). Serum BDNF concentrations as peripheral manifestations of depression: evidence from a systematic review and meta-analyses on 179 associations (*N* = 9484). *Mol. Psychiatry* 19 791–800. 10.1038/mp.2013.105 23958957

[B16] Nargis T. Chakrabarti P. (2018). Significance of circulatory DPP4 activity in metabolic diseases. *IUBMB Life* 70 112–119. 10.1002/iub.1709 29331088

[B17] Nasreddine Z. S. Phillips N. A. Bedirian V. Charbonneau S. Whitehead V. Collin I. (2005). The montreal cognitive assessment, MoCA: a brief screening tool for mild cognitive impairment. *J. Am. Geriatr. Soc.* 53 695–699. 10.1111/j.1532-5415.2005.53221.x 15817019

[B18] Ooi C. P. Loke S. C. Yassin Z. Hamid T. A. (2011). Carbohydrates for improving the cognitive performance of independent-living older adults with normal cognition or mild cognitive impairment. *Cochrane Database Syst. Rev.* 4:CD007220. 10.1002/14651858.CD007220.pub2 21491398 PMC7388979

[B19] Papathanassoglou E. D. Miltiadous P. Karanikola M. N. (2015). May BDNF be implicated in the exercise-mediated regulation of inflammation? critical review and synthesis of evidence. *Biol. Res. Nurs.* 17 521–539. 10.1177/1099800414555411 25358684

[B20] Petersen R. C. Roberts R. O. Knopman D. S. Boeve B. F. Geda Y. E. Ivnik R. J. (2009). Mild cognitive impairment: ten years later. *Arch. Neurol.* 66 1447–1455. 10.1001/archneurol.2009.266 20008648 PMC3081688

[B21] Qin X. Y. Cao C. Cawley N. X. Liu T. T. Yuan J. Loh Y. P. (2017). Decreased peripheral brain-derived neurotrophic factor levels in Alzheimer’s disease: a meta-analysis study (N = 7277). *Mol. Psychiatry.* 22 312–320. 10.1038/mp.2016.62 27113997

[B22] Shah Z. Kampfrath T. Deiuliis J. A. Zhong J. Pineda C. Ying Z. (2011). Long-term dipeptidyl- peptidase 4 inhibition reduces atherosclerosis and inflammation via effects on monocyte recruitment and chemotaxis. *Circulation* 124 2338–2349. 10.1161/CIRCULATIONAHA.111.041418 22007077 PMC4224594

[B23] Tegeler C. O’Sullivan J. L. Bucholtz N. Goldeck D. Pawelec G. Steinhagen-Thiessen E. (2016). The inflammatory markers CRP, IL-6, and IL-10 are associated with cognitive function–data from the Berlin aging study II. *Neurobiol. Aging* 38 112–117. 10.1016/j.neurobiolaging.2015.10.039 26827649

[B24] Tian L. Gao J. Hao J. Zhang Y. Yi H. O’Brien T. D. (2010). Reversal of new-onset diabetes through modulating inflammation and stimulating beta-cell replication in nonobese diabetic mice by a dipeptidyl peptidase IV inhibitor. *Endocrinology* 151 3049–3060. 10.1210/en.2010-0068 20444936

[B25] Trajkovska V. Marcussen A. B. Vinberg M. Hartvig P. Aznar S. Knudsen G. M. (2007). Measurements of brain-derived neurotrophic factor: methodological aspects and demographical data. *Brain Res. Bull.* 73 143–149. 10.1016/j.brainresbull.2007.03.009 17499648

[B26] Weaver J. D. Huang M. H. Albert M. Harris T. Rowe J. W. Seeman T. E. (2002). Interleukin-6 and risk of cognitive decline: MacArthur studies of successful aging. *Neurology* 59 371–378. 12177370 10.1212/wnl.59.3.371

[B27] Wu A. Ying Z. Gomez-Pinilla F. (2004). The interplay between oxidative stress and brain-derived neurotrophic factor modulates the outcome of a saturated fat diet on synaptic plasticity and cognition. *Eur. J. Neurosci.* 19 1699–1707. 10.1111/j.1460-9568.2004.03246.x 15078544

[B28] Yang L. Song J. Zhang X. Xiao L. Hu X. Pan H. (2018). Association of serum angiopoietin-like protein 8 with albuminuria in type 2 diabetic patients: results from the GDMD study in China. *Front. Endocrinol.* 9:414. 10.3389/fendo.2018.00414 30072957 PMC6058027

[B29] Yisireyili M. Takeshita K. Hayashi M. Wu H. Uchida Y. Yamamoto K. (2016). Dipeptidyl peptidase- IV inhibitor alogliptin improves stress-induced insulin resistance and prothrombotic state in a murine model. *Psychoneuroendocrinology* 73 186–195. 10.1016/j.psyneuen.2016.08.004 27509090

[B30] Zhang X. Zhang Z. Yang Y. Suo Y. Liu R. Qiu J. (2018). Alogliptin prevents diastolic dysfunction and preserves left ventricular mitochondrial function in diabetic rabbits. *Cardiovasc. Diabetol.* 17:160. 10.1186/s12933-018-0803-z 30591063 PMC6307280

[B31] Zheng T. Baskota A. Gao Y. Chen T. Tian H. Yang F. (2015a). Increased plasma DPP4 activities predict new-onset hyperglycemia in Chinese over a four-year period: possible associations with inflammation. *Metabolism* 64 498–505. 10.1016/j.metabol.2014.12.004 25592717

[B32] Zheng T. Chen T. Liu Y. Gao Y. Tian H. (2015b). Increased plasma DPP4 activity predicts new-onset hypertension in Chinese over a 4-year period: possible associations with inflammation and oxidative stress. *J. Hum. Hypertens.* 29 424–429. 10.1038/jhh.2014.111 25411054

[B33] Zheng T. P. Liu Y. H. Yang L. X. Qin S. H. Liu H. B. (2015c). Increased plasma dipeptidyl peptidase-4 activities are associated with high prevalence of subclinical atherosclerosis in Chinese patients with newly diagnosed type 2 diabetes: a cross-sectional study. *Atherosclerosis* 242 580–588. 10.1016/j.atherosclerosis.2015.07.042 26318108

[B34] Zheng T. Chen B. Yang L. Hu X. Zhang X. Liu H. (2017). Association of plasma dipeptidyl peptidase-4 activity with non-alcoholic fatty liver disease in nondiabetic Chinese population. *Metabolism* 73 125–134. 10.1016/j.metabol.2017.04.012 28637594

[B35] Zheng T. Gao Y. Baskota A. Chen T. Ran X. Tian H. (2014). Increased plasma DPP4 activity is predictive of prediabetes and type 2 diabetes onset in Chinese over a four-year period: result from the China national diabetes and metabolic disorders study. *J. Clin. Endocrinol. Metab.* 99 E2330–E2334. 10.1210/jc.2014-1480 25029421

[B36] Zheng T. Ge B. Liu H. Chen B. Qin L. Xiao L. (2018a). Triglyceride-mediated influence of serum angiopoietin-like protein 8 on subclinical atherosclerosis in type 2 diabetic patients: results from the GDMD study in China. *Cardiovasc. Diabetol.* 17:84. 10.1186/s12933-018-0687-y 30007407 PMC6046091

[B37] Zheng T. Liu H. Qin L. Chen B. Zhang X. Hu X. (2018b). Oxidative stress-mediated influence of plasma DPP4 activity to BDNF ratio on mild cognitive impairment in elderly type 2 diabetic patients: results from the GDMD study in China. *Metabolism* 87 105–112. 10.1016/j.metabol.2018.03.014 29572131

[B38] Zheng T. Liu Y. Qin S. Liu H. Yang L. Zhang X. (2016a). Increased dipeptidyl peptidase-4 activity is associated with high prevalence of depression in middle-aged and older adults: a cross-sectional study. *J. Clin. Psychiatry* 77 e1248–e1255. 10.4088/JCP.15m10154 27529287

[B39] Zheng T. Qin L. Chen B. Hu X. Zhang X. Liu Y. (2016b). Association of plasma dpp4 activity with mild cognitive impairment in elderly patients with type 2 diabetes: results from the gdmd study in China. *Diabetes Care* 39 1594–1601. 10.2337/dc16-0316 27371673

[B40] Zhong J. Rajagopalan S. (2015). Dipeptidyl peptidase-4 regulation of SDF-1/CXCR4 axis: implications for cardiovascular disease. *Front. Immunol.* 6:477. 10.3389/fimmu.2015.00477 26441982 PMC4585326

[B41] Zhong J. Rao X. Deiuliis J. Braunstein Z. Narula V. Hazey J. (2013). A potential role for dendritic cell/macrophage-expressing DPP4 in obesity-induced visceral inflammation. *Diabetes* 62 149–157. 10.2337/db12-0230 22936179 PMC3526020

