# Supplementary figures and images for: The Relationship Between Plasma DPP4 Activity to BDNF Ratio and Mild Cognitive Impairment in Elderly Population With Normal Glucose Tolerance
Source: Front Aging Neurosci. 2019 Mar 4;11:33. doi: 10.3389/fnagi.2019.00033 (PMC6409327; doi:10.3389/fnagi.2019.00033)

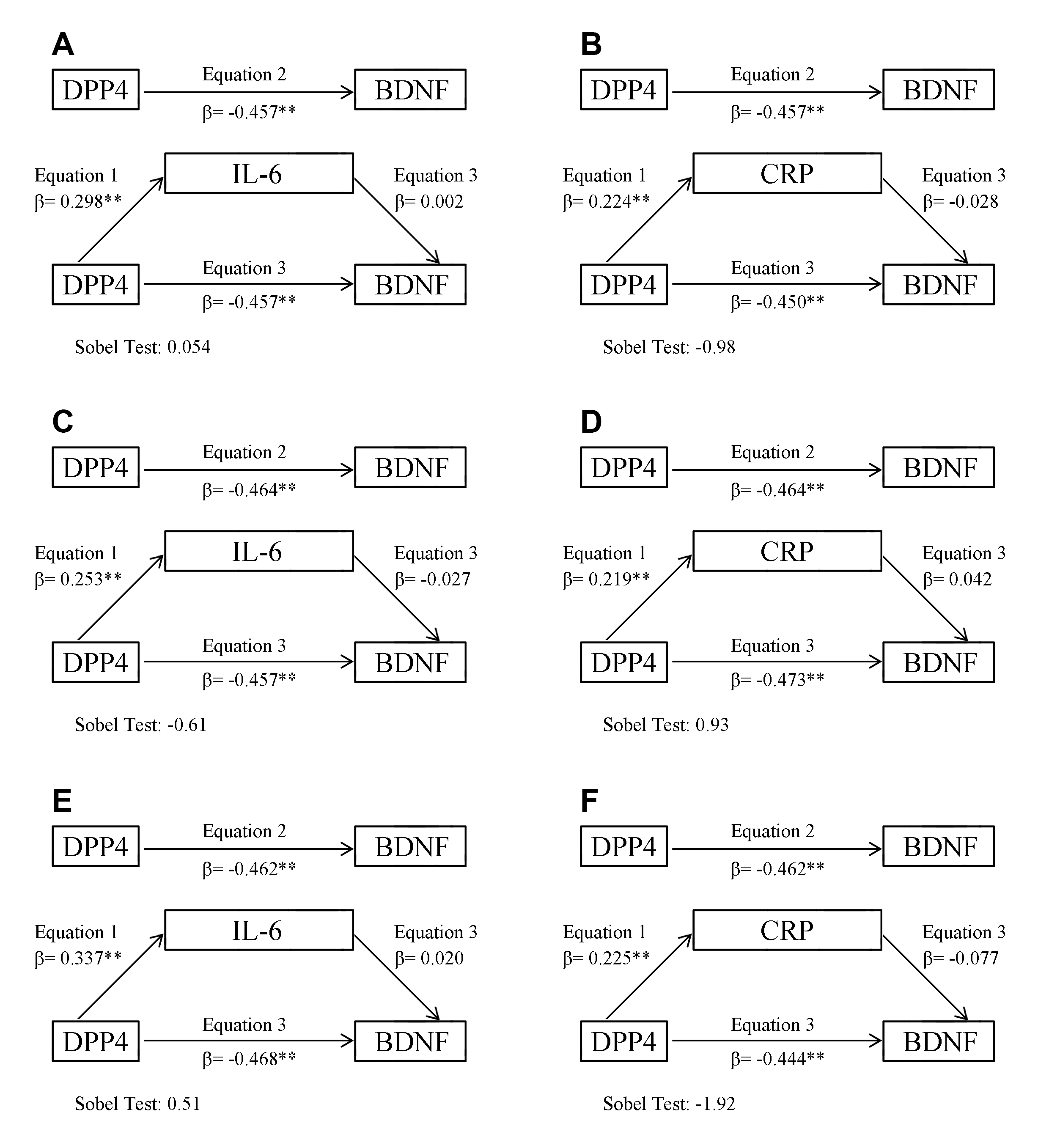

Supplement: FIGURE S1 — Inflammation mediation models of the relationship between plasma DPP4 activity and BDNF (A) IL-6 mediation models of the relationship between plasma DPP4 activity and BDNF in all participants. (B) CRP mediation models of the relationship between plasma DPP4 activity and BDNF in all participants. (C) IL-6 mediation models of the relationship between plasma DPP4 activity and BDNF in men. (D) CRP mediation models of the relationship between plasma DPP4 activity and BDNF in men. (E) IL-6 mediation models of the relationship between plasma DPP4 activity and BDNF in women. (F) CRP mediation models of the relationship between plasma DPP4 activity and BDNF in women. ∗P < 0.05, ∗∗P < 0.01. [file Image_1.JPEG]

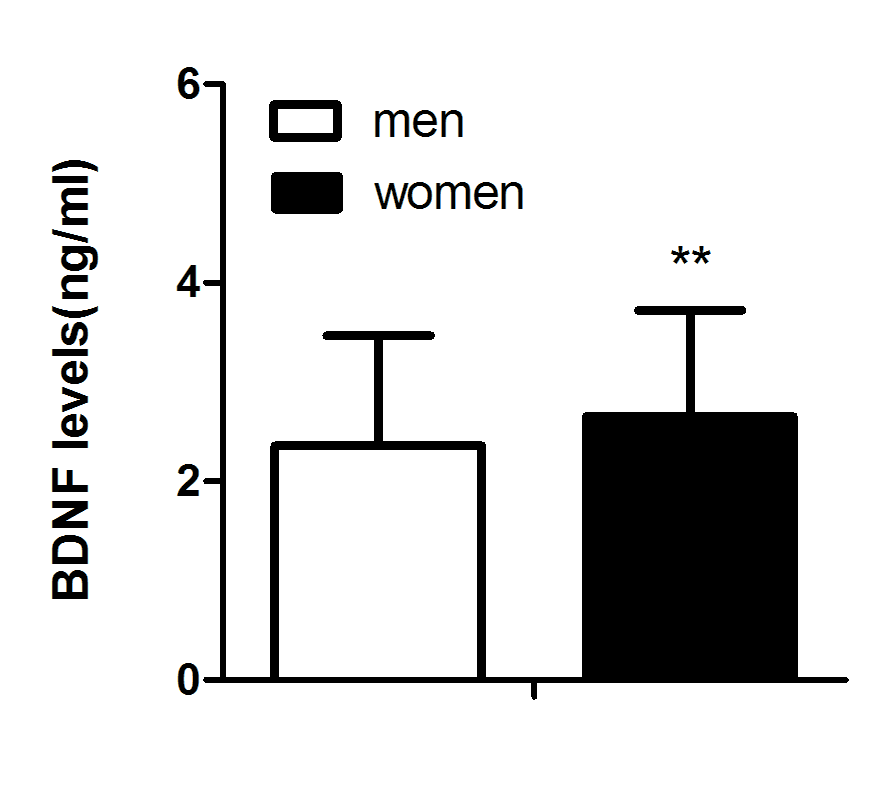

Supplement: FIGURE S2 — Comparison of BDNF levels between men and women. ∗P < 0.05, ∗∗P < 0.01. [file Image_2.TIF]
