# Supplementary material for: The Relationship Between Plasma DPP4 Activity to BDNF Ratio and Mild Cognitive Impairment in Elderly Population With Normal Glucose Tolerance
Source: Front Aging Neurosci. 2019 Mar 4;11:33. doi: 10.3389/fnagi.2019.00033 (PMC6409327; doi:10.3389/fnagi.2019.00033)
Supplement: TABLE S1 — Characteristics of the 1066 participants by MCI. [file Table_1.DOC]

Supplementary table1. Characteristics of the 1066 participants by MCI.

|  | Without MCI(control) | With MCI | |  |
| --- | --- | --- | --- | --- |
|  |  |  |  | P Value |
| n | 897 | | 169 | - |
| Age (years) | 68.5±5.4 | | 70.6±5.5 | <0.001 |
| Percent men (%) | 41.1 | | 45.6 | 0.285 |
| BMI (kg/m^2^) | 23.2±3.7 | | 23.9±3.9 | 0.016 |
| Current smoking (%) | 19.8 | | 21.9 | 0.542 |
| Habitual alcohol drinking (%) | 16.9 | | 18.3 | 0.658 |
| Leisure-time physical  activity (%) | 57.3 | | 54.4 | 0.490 |
| Education level |  | |  | 0.004 |
| ≦Primary school | 46.9 | | 59.8 |  |
| Middle school | 43.8 | | 36.1 |  |
| ≥High school | 9.3 | | 4.1 |  |
| Annual income, RMB |  | |  | 0.810 |
| ≦5000 | 4.8 | | 4.7 |  |
| 5000-30000 | 45.8 | | 43.2 |  |
| >30000 | 49.4 | | 52.1 |  |
| Statin use (%) | 10.7 | | 14.2 | 0.187 |
| NSAID use (%) | 6.0 | | 7.1 | 0.593 |
| Cardiovascular disease (%) | 7.7 | | 8.9 | 0.600 |
| IL-6(pg/ml) ^a^ | 1.35(1.11, 1.58) | | 1.42(1.12, 1.73) | 0.275 |
| CRP(mg/L) ^a^ | 1.16(0.94, 1.34) | | 1.18(0.96,1.51) | 0.072 |
| Nitrotyrosine (µmol/L) ^a^ | 0.38±0.12 | | 0.43±0.14 | <0.001 |
| 8-iso-PGF2a (pg/mL) ^a^ | 41.7±10.5 | | 46.6±11.1 | <0.001 |
| DPP4 activity(nmol/min/ml) ^a^ | 17.5±8.0 | | 21.2±7.9 | <0.001 |
| BDNF(ng/ml)^a^ | 2.62±1.09 | | 2.05±1.04 | <0.001 |
| MoCA score ^a^ | 27.7±1.3 | | 22.4±1.8 | <0.001 |
| DBR ^a^ | 9.23±9.10 | | 14.51±11.17 | <0.001 |

^a^ Adjusted for age, gender, and BMI.
